# A dataset on multi-trait selection approaches for screening desirable wild relatives of wheat

**DOI:** 10.1016/j.dib.2021.107541

**Published:** 2021-11-03

**Authors:** Alireza Pour-Aboughadareh, Peter Poczai

**Affiliations:** a Seed and Plant Improvement Institute, Agricultural Research, Education and Extension Organization (AREEO), Karaj 3183964653, Iran; b Botany Unit, Finnish Museum of Natural History, University of Helsinki, P.O. Box 7, Helsinki FI‐00014, Finland

**Keywords:** Crop wild relatives, Drought stress, MGIDI index, Physiological trait, Root system features

## Abstract

Wild relatives of common wheat are an extraordinary source of tolerance to various environmental stresses. The dataset herein presents the effect of water-deficit stress on a core collection of landraces and wild relatives of wheat (including 180 samples belonging to four *Triticum* and eight *Aegilops* species [*T. boeoticum* Bioss., *T. urartu* Gandilyan., *T. durum* Def., *T. aestivum* L., *Ae. speltoides* Tausch., *Ae. tauschii* Coss., *Ae. caudata* L., *Ae. umbellulata* Zhuk., *Ae. neglecta* L., *Ae. cylindrica* Host., *Ae. crassa* Boiss., and *Ae. triuncialis*]) in terms of several physiological traits, root and shoot biomasses, and features of root system architecture (RSA). All genetic materials were subjected to water-stress treatment using a pot experiment under greenhouse conditions. To screen the most tolerant accessions, three selection indices, such as Smith and Hazel (SH), factor analysis and ideotype‐design (FAI), and the multi-trait genotype-ideotype distance index (MGIDI) were computed. The obtained data can highlight the role of some features of RSA in increasing water-deficit tolerance in some wild relatives of wheat. Moreover, the use of selection indices in the early stage of growth can be highlighted for future research.

## Specifications Table

**Table:** 

Subject	Data analysis (Agricultural and Biological Science)
Specific subject area	Agronomy and Crop Science
Type of data	Tables and Figures
How data were acquired	All data were collected from pot experiments under controlled greenhouse conditions on a set of wild relatives of wheat belonging to 12 Triticum and Aegilops species, such as *T. boeoticum* Bioss., *T. urartu* Gandilyan., *T. durum* Def., *T. aestivum* L., *Ae. speltoides* Tausch., *Ae. tauschii* Coss., *Ae. caudata* L., *Ae. umbellulata* Zhuk., *Ae. neglecta* L., *Ae. cylindrica* Host., *Ae. crassa* Boiss., and *Ae. triuncialis*. Data presented in tables and figures were obtained by measuring a set of physiological traits and some features related to root system architecture (RSA) under two control and water deficit stress conditions. Three selection indices were used to rank the accessions based on information of multiple traits. All data were subjected to statistical analysis using package ‘metan’ in R software.
Data format	Raw and analyzed
Parameters for data collection	A controlled greenhouse condition was used to collect the dataset. The experimental data consisted of a set of features related to root system architecture (RSA) and physiological traits including root fresh weight (RFW), root dry weight (RDW), root tissue density (RTD), specific root length (SRL), root diameter (RD), root branch number (RBN), root surface area (RA), total root surface area (TSA), root volume (RV), shoot fresh weight (SFW), shoot dry weight (SDW), shoot-to-root fresh weight ratio (SRF), shoot-to-root dry weight ratio (SRD), relative chlorophyll content (SPAD), leaf temperature (LT), stomatal conductance (Gs), initial fluorescence (Fo), maximum fluorescence (Fm), maximum quantum yield of PSII (Fv/Fm), maximum primary yield of photochemistry of PSII (Fv/Fo), relative water content (RWC), shoot fresh weight (SFW), and shoot dry weight (SDW).
Description of data collection	A greenhouse pot experiment was performed in 2015–2016 at the Crop Production and Breeding Department, Imam Khomeini International University, Qazvin, Iran. Five seeds from each accession were planted into plastic pots (40-cm height and 20-cm diameter) filled with a mixture of dry soil and sand in a ratio of 3:1 (2 kg). All pots were arranged in a factorial experiment based on a randomized complete block with two replications under an optimal growing photoperiod (16/8 h light/dark cycle) and temperature (25/20°C day/night). The water-deficit treatment was determined based on a method proposed by Souza et al. (2000) and started at the three-leaf stage of seedling growth. Seedling plants were sampled after 30 days of stress treatment. At this stage of experiment, 23 traits belonging to physiological, root phenology, and biomass features were recorded on seedling plants.
Data source location	Department of Crop Production and Breeding, Imam Khomeini International University, Qazvin, Iran.
Data accessibility	The raw data associated to this article are provided on Mendeley dataset http://dx.doi.org/10.17632/84kmmmgvvr.1
Related research article	[1] A. Pour-Aboughadareh, J. Ahmadi, A.A. Mehrabi, A. Etminan, M. Moghaddam, K.H.M. Siddique. Physiological responses to drought stress in wild relatives of wheat: implications for wheat improvement, Acta Physiol. Plant. 39(2017) 106. https://doi.org/10.1007/s11738-017-2403-z

## Value of the Data

• The dataset analyzed in the current report reveals an overview of some wild wheat species for improvement of drought tolerance in wheat due to their potential in response to severe water deficit stress conditions.
• This dataset indicates that root system features have a significant role in the discrimination of wild relatives of wheat under water-deficit conditions.
• Our data can highlight the applicability of the selection indices, especially MGIDI index, in selecting the best plant genetic materials based on multi-trait assessment in the early growth stage.
• As a remark conclusion, these data provide a new insight into use of the evaluated wheat genetic resource for discovering new agronomic features and even new drought-related genes from alien genome for transfer in bred wheat variety.

## 1 Data Description

Wild relatives of wheat serve as an important gene pool for any wheat breeding program, due to their potential to confer useful features to modern genotypes [2]. Two genera, *Aegilops* and *Triticum*, have been identified as the main germplasm of wheat and together consist of 27 wild species [3]. As reported in numerous studies, each wild relative of wheat has at least one ideal feature, such as resistance to various biotic and abiotic stresses [4], [5], [6], [7], [8], [9]. Recently, Pour-Aboughadareh et al. [10] reviewed the potential of different wild wheat species in terms of various biotic and abiotic stresses and indicated that these gene pools how to improve the genetic basis of the bred genotypes. Among the abiotic stresses, drought is the most relevant stress affecting plant growth and production in large parts of the world [1]. Climate change in recent years has dramatically affected wheat production globally. On the other hand, the narrowing of the genetic basis of improved bread wheat cultivars is another important issue that influences breeding programs. Hence, assessing the genetic diversity and exploring ideal accessions among the wild relatives can provide new insights toward further conservation and utilization of these relatives.

Plant breeders often try to pyramid various suitable agronomic features in one superior genotype that finally leads to achieving high performance. In this regard, several selection indices up to now have been suggested to select superior genotypes. The proposed indices such as Smith-Hazel (SH) [11,12], and factor analysis and ideotype‐design [13], restrict breeders in selection the best genotypes due to their some limitations like expressing the economic values and converting them into realistic economic weightings [14]. To overcome these limitations, recently the multi-trait index based on factorial analysis and genotype-ideotype distance index (MGIDI) was suggested by Olivoto and Nardino [13]. Indeed MGIDI focuses on the selection of best genotypes where multiple traits have been measured. The use efficiency of this index in identifying the superior genotypes was also reported in several studies [15], [16], [17].

**Fig. 1:**
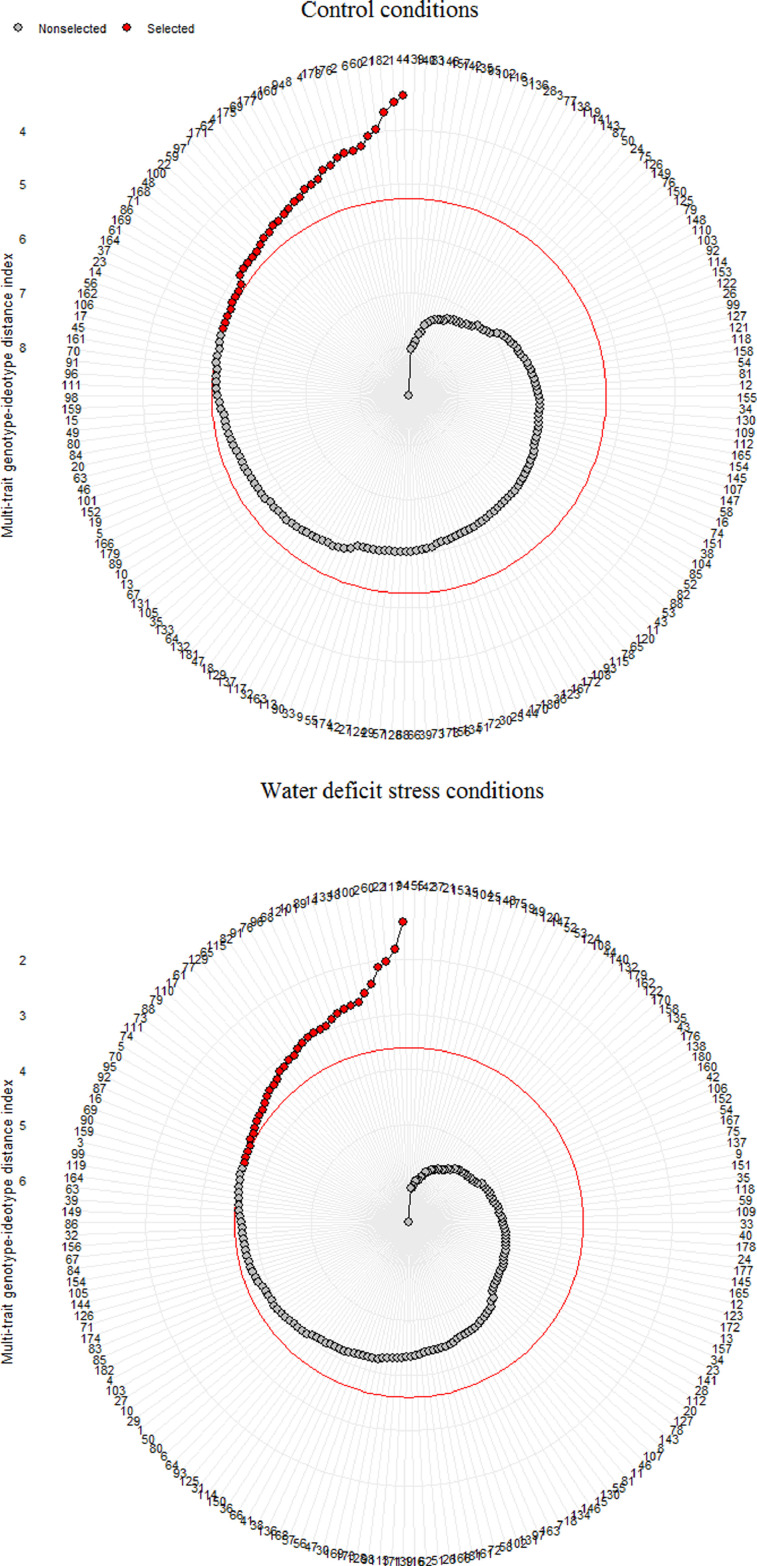
Accession ranking in ascending order for the MGIDI index. The selected accessions based on MGIDI index are shown in red. The central red circle represents the cut point according to the selection pressure. See Table 3 for selected accessions.

The dataset is presented in four tables and one figure that describe the usefulness of multivariate selection indices in identifying desirable wild wheat accessions under two growth conditions. Table 3 shows the filtered measured traits that have significant effects on genetic diversity among the 180 investigated accessions. Based on this table, the discriminator traits under control conditions were root and shoot fresh weights (RFW and SFW), root tissue density (RTD), root surface area (RA), total root surface area (TSA), root volume (RV), shoot-to-root fresh weight ratio (SRF), relative chlorophyll content (SPAD index), maximum fluorescence (Fm), and initial fluorescence (Fo). Under water-deficit conditions, these traits were specific root length (SRL), RA, RSA, root branch number (RBN), RV, SPAD, leaf temperature (LT), stomatal conductance (Gs), relative water content (RWC), Fm, and Fo. Fig. 1 indicates the result of screening the investigated plant genetic accessions based on MGIDI index. In this figure, the red circle indicates the cut point according to the selection pressure (SI = 20%). The MGIDI index identified 36 samples as more desirable accessions than others for each growth condition. Among these, accession numbers 1, 14, 22, 48, 60, 61, 69, 94, and 100 were selected in both conditions, suggesting that they can maintain their ideal growth under both conditions. Considering the results presented in Table 3, the selected accessions have breeding potential in terms of RA, SRA, RV, SPAD, Fm, and Fo features.

**Fig. 2:**
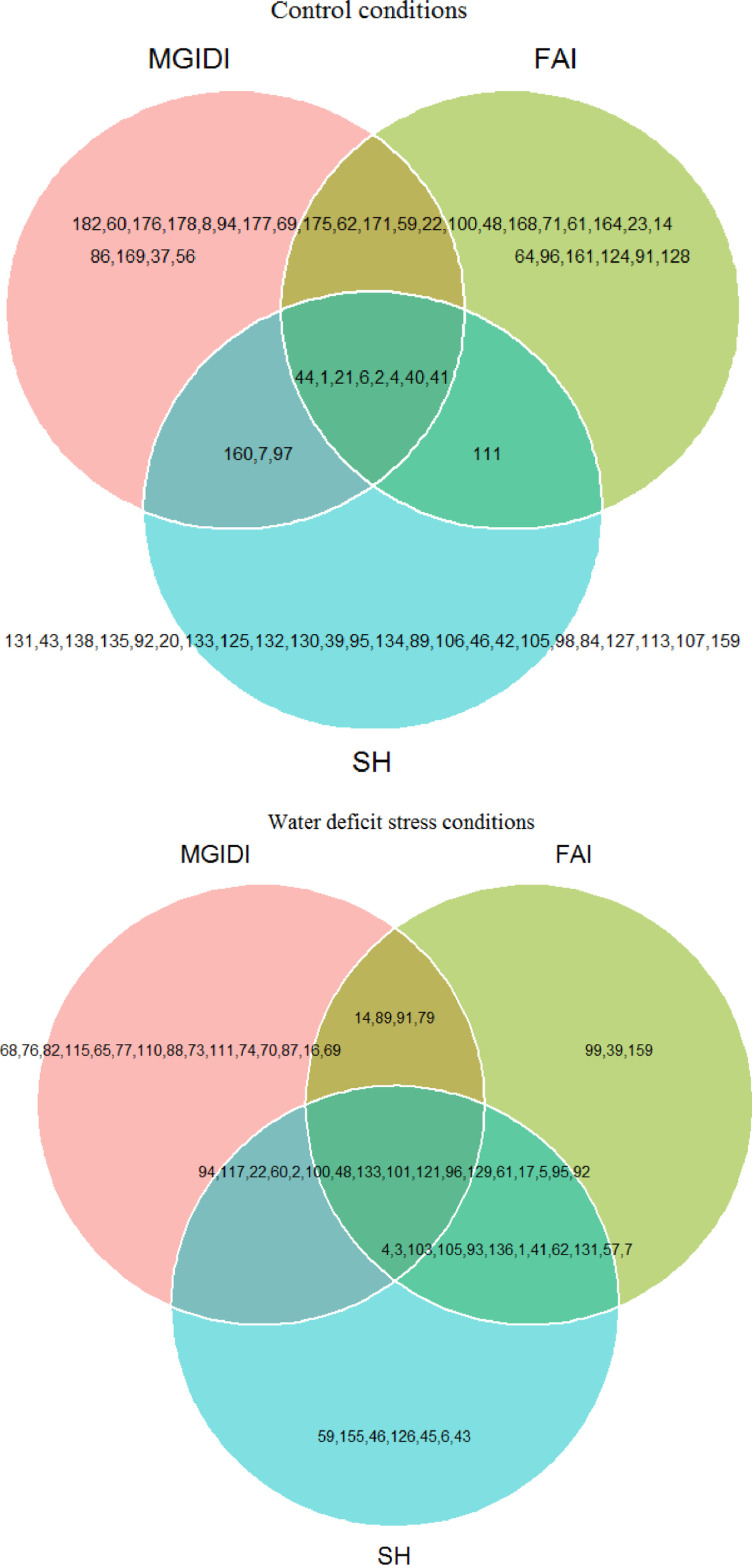
Venn plot indicating the relationships between the MGIDI, FAI, and SH indexes under control and water deficit stress conditions.

**Table 1:** List of the evaluated Iranian *Aegilops* and *Triticum* species in this work.

Species	Genome	Samples
*T. aestivum* (ABD)	AABBD	1–19
*T. boeoticum* (A^b^)	A^b^A^b^	20–36
*T. durum* (AB)	AABB	37–54
*T. urartu* (A^u^)	A^u^A^u^	55–71
*Ae. caudata* (C)	CC	72–78
*Ae. crassa* (DM)	DDMM	79–92
*Ae. cylindrica* (DC)	DDCC	93–111
*Ae. neglecta* (UM)	UUMM	112–122
*Ae. speltoides* (S)	SS	123–128
*Ae. tauschii* (D)	DD	129–148
*Ae. triuncialis* (CU)	CCUU	149–163
*Ae. umbellulata* (U)	UU	164–180

**Table 2:** Description of root and shoot biomasses, physiological traits, and root system architecture system features in a wheat core collection grown under two control and water deficit stress conditions.

Trait	Abb.	unit	Refs.
Root fresh weight	RFW	mg plant^–1^	[1]
Root dry weight	RDW	mg plant^–1^	[1]
Root tissue density	RTD	mg cm ^–3^	[4]
Specific root length	SRL	m g^–1^ dry mass	[4]
Root diameter	RD	cm	[4]
Root branch number	RBN		[4]
Root surface area	RA	cm^2^	[4]
Total root surface area	TSA	cm^2^	[4]
Root volume	RV	cm^3^	[1]
Shoot fresh weight	SFW	mg plant^–1^	[1]
Shoot dry weight	SDW	mg plant^–1^	[4]
Shoot-to-root fresh weight ratio	SRF		[4]
Shoot-to-root dry weight ratio	SRD		[1]
Relative chlorophyll content	SPAD	SPAD value	[1]
Leaf temperature	LT	°C	[1]
Stomatal conductance	Gs	mmol m^−2^ s^−1^	[1]
Initial fluorescence	Fo		[1]
Maximum fluorescence	Fm		[1]
Maximum quantum yield of PSII	Fv/Fm		[1]
Maximum primary yield of photochemistry of PSII	Fv/Fo		[1]
Relative water content	RWC	%	[1]
Shoot fresh weight	SFW	mg plant^–1^	[1]
Shoot dry weight	SDW	mg plant^–1^	[1]

**Table 3:** Significant discriminator traits for screening desirable accessions in each selection indices under control and water deficit stress conditions.

Trait	Abb.	Control conditions	Water deficit conditions
Root fresh weight	RFW	✓	
Root dry weight	RDW		
Root tissue density	RTD	✓	
Specific root length	SRL		✓
Root diameter	RD		
Root branch number	RBN		✓
Root surface area	RA	✓	✓
Total root surface area	TSA	✓	✓
Root volume	RV	✓	✓
Shoot fresh weight	SFW	✓	
Shoot dry weight	SDW		
Shoot-to-root fresh weight ratio	SRF	✓	
Shoot-to-root dry weight ratio	SRD		
Relative chlorophyll content	SPAD	✓	✓
Leaf temperature	LT		✓
Stomatal conductance	Gs		✓
Initial fluorescence	Fo	✓	✓
Maximum fluorescence	Fm	✓	✓
Maximum quantum yield of PSII	Fv/Fm		
Maximum primary yield of photochemistry of PSII	Fv/Fo		
Relative water content	RWC		✓
Shoot fresh weight	SFW		
Shoot dry weight	SDW

**Table 4:** Selected desirable accessions using each selection index under control and water deficit stress conditions.

Control conditions			Water deficit stress conditions		
MGIDI	FAI	SH	MGIDI	FAI	SH
1 (TA)	1 (TA)	1 (TA)	2 (TA)	1 (TA)	1 (TA)
2 (TA)	2 (TA)	2 (TA)	5 (TA)	2 (TA)	2 (TA)
4 (TA)	4 (TA)	4 (TA)	6 (TA)	3 (TA)	3 (TA)
6 (TA)	6 (TA)	6 (TA)	14 (TA)	4 (TA)	4 (TA)
7 (TA)	8 (TA)	20 (TB)	16 (TA)	5 (TA)	5 (TA)
8 (TA)	14 (TA)	21 (TB)	17 (TA)	6 (TA)	6 (TA)
14 (TA)	21 (TB)	39 (TD)	22 (TB)	7 (TA)	7 (TA)
21 (TB)	22 (TB)	40 (TD)	48 (TD)	14 (TA)	17 (TA)
22 (TB)	23 (TB)	41 (TD)	61 (TU)	15 (TA)	22 (TB)
23 (TB)	40 (TD)	42 (TD)	65 (TU)	17 (TA)	41 (TD)
37 (TD)	41 (TD)	43 (TD)	68 (TU)	22 (TB)	43 (TD)
40 (TD)	44 (TD)	44 (TD)	69 (TU)	39 (TD)	45 (TD)
41 (TD)	48 (TD)	46 (TD)	70 (TU)	41 (TD)	46 (TD)
44 (TD)	59 (TU)	84 (AC)	73 (ACA)	48 (TD)	48 (TD)
48 (TD)	60 (TU)	89 (AC)	74 (ACA)	57 (TU)	57 (TU)
56 (TU)	61 (TU)	92 (AC)	76 (ACA)	61 (TU)	59 (TU)
59 (TU)	62 (TU)	95 (ACR)	77 (ACA)	62 (TU)	61 (TU)
60 (TU)	64 (TU)	97 (ACR)	79 (AC)	79 (AC)	62 (TU)
61 (TU)	69 (TU)	98 (ACR)	82 (AC)	89 (AC)	92 (AC)
62 (TU)	71 (TU)	105 (ACR)	87 (AC)	91 (AC)	93 (AC)
69 (TU)	91 (AC)	106 (ACR)	88 (AC)	92 (AC)	94 (AC)
71 (TU)	94 (ACY)	107 (ACR)	89 (AC)	93 (ACY)	95 (AC)
86 (AC)	96 (ACY)	111 (ACR)	91 (AC)	94 (ACY)	96 (AC)
94 (ACY)	100 (ACY)	113 (AN)	92 (AC)	95 (ACY)	100 (AC)
97 (ACY)	111 (ACY)	125 (AS)	94 (ACY)	96 (ACY)	101 (AC)
100 (ACY)	124 (AS)	127 (AS)	95 (ACY)	99 (ACY)	103 (AC)
160 (ATR)	128 (AS)	130 (AT)	96 (ACY)	100 (ACY)	105 (AC)
164 (AU)	161 (ATR)	131 (AT)	100 (ACY)	101 (ACY)	117 (AN)
168 (AU)	164 (AU)	132 (AT)	101 (ACY)	103 (ACY)	121 (AN)
169 (AU)	168 (AU)	133 (AT)	110 (ACY)	105 (ACY)	126 (AS)
171 (AU)	171 (AU)	134 (AT)	111 (ACY)	117 (AN)	129 (AT)
175 (AU)	175 (AU)	135 (AT)	115 (AN)	121 (AN)	131 (AT)
176 (AU)	176 (AU)	138 (AT)	117 (AN)	129 (AT)	133 (AT)
177 (AU)	177 (AU)	159 (ATR)	121 (AN)	131 (AT)	136 (AT)
178 (AU)	178 (AU)	160 (ATR)	129 (AT)	133 (AT)	155 (AT)
182 (AU)	182 (SC)		133 (AT)	136 (AT)	

MGIDI, multi-trait genotype-ideotype distance index; FAI, factor analysis and ideotype‐design; SH, Smith and Hazel

TA, T. *aestivum*; TB, *T. boeoticum*; TD, *T. durum*; TU, *T. urartu*; ACA, *Ae. caudata*; AC, *Ae. crassa*; ACY, *Ae. cylindrica*; AN, *Ae. neglecta*; AS, *Ae. speltoides*; AT, *Ae. tauschii*; ATR, *Ae. triuncialis*; AU, *Ae. umbellulata*.

Table 4 presents the selected accessions based on Smith-Hazel (SH), factor analysis and ideotype‐design (FAI), and MGIDI indices. Fig. 2 revealed a Venn plot for the selected accessions based on three selection indices. Under control conditions, all three selection indices together identified accession numbers 1, 2, 4, 6, 21, 40, 41, and 44 as the best accessions. Under water-deficit conditions, accession numbers 2, 5, 17, 22, 48, 60, 61, 92, 94, 95, 96, 100, 101, 117, 121, 129, and 131 were selected as the best accessions with desirable root-system features and some physiological traits.

## 2 Experimental Design, Materials and Methods

### 2.1 Plant materials

A set of 180 wild relatives of wheat and landraces belonging to four species of *Triticum* genus (*T. aestivum* L., *T. durum* Def., *T. boeoticum* Bioss., and *T. urartu* Gandilyan.) and eight species of Aegilops genus (*Ae. speltoides* Tausch., *Ae. tauschii* Coss., *Ae. caudata* L., *Ae. umbellulata* Zhuk., *Ae. neglecta* L., *Ae. cylindrica* Host., *Ae. crassa* Boiss., and *Ae. triuncialis*) were investigated under control and water deficit stress conditions (Table 1). All genetic materials were provided from Ilam University Genebank (IUGB).

### 2.2 Experimental design

A pot experiment was performed at the Crop Production and Breeding Department, Imam Khomeini International University, Qazvin, Iran. Each plastic pot (20 cm diameter, 40 cm height) was filled with 2 kg of dry soil and sand in a ratio of 3:1. Five seeds from each accession were planted into each plastic pot and were maintained at an optimal growing photoperiod (16/8 h light/dark cycle) and temperature (25/20°C day/night) conditions. After seed germination and seedling establishment, the pots were arranged in a factorial experiment based on a randomized complete block design (RCBD) with two replications. The water deficit stress treatment was initiated at the three-leaf stage of seedling growth using the field capacity (FC) method as proposed by Souza et al. [18]. Accordingly, half of the seedling plants were maintained under full FC (as the control conditions) and the other half were subjected to 30% FC for 30 days (as the stress conditions). Seedlings were sampled after 30 days of stress treatment*.*

### 2.3 Data collection

After 30 days after stress treatment, seedling plants were sampled and 23 traits were measured. The list of measured traits along with their units is given in Table 2.

### 2.4 Statistical analysis

Three selection indices, including multi-trait genotype-ideotype distance index (MGIDI), Smith-Hazel (SH), and factor analysis and ideotype‐design (FAI) were used to select the desirable accessions in terms of a complex root and physiological traits. All analyses were computed in R software using the ‘metan’ package [19].

## Ethics Statement

This manuscript is not currently being considered for publication elsewhere.

## CRediT authorship contribution statement

**Alireza Pour-Aboughadareh:** Conceptualization, Methodology, Software, Data curation, Writing – original draft, Investigation. **Peter Poczai:** Visualization, Writing – review & editing.

## Declaration of Competing Interest

The authors declare that they have no known competing financial interests or personal relationships that have, or could be perceived to have, influenced the work reported in this manuscript.
